# Choosing to Implement Value-Based Healthcare Initiatives: A Strategic Decision for Achieving Better Performance in Improving Population Health: Comment on "Reflections on Managing the Performance of Value-Based Healthcare: A Scoping Review"

**DOI:** 10.34172/ijhpm.9050

**Published:** 2025-04-16

**Authors:** Ana Paula Beck da Silva Etges

**Affiliations:** ^1^National Institute of Science and Technology for Health Technology Assessment (IATS), Porto Alegre, Brazil.; ^2^PEV Healthcare Consulting, Porto Alegre, Brazil.; ^3^Programa de Pós-graduação em Epidemiologia da Escola de Medicina da Universidade Federal do Rio Grande do Sul, Porto Alegre, Brazil.

**Keywords:** Value-Based Healthcare, Strategy, Operational Management, Value in Health

## Abstract

Operational effectiveness is about improving what is being done, reducing errors and harms, and improving efficiency, while strategy involves making decisions and choices. Implementing value-based healthcare (VBHC) also means matching previous strategies and performance literature to guide building sustainable organizations in healthcare businesses. This commentary paper explores answers for: What does it mean to have a high-performance, sustainable, and impactful health organization? By describing frameworks about leadership and social capital, this piece argues that the healthcare system’s sustainability involves making choices that set as a strategy implementing VBHC principles, cause implications on regulatory, organizational, and individual levels, and result in structuring systems that contribute to achieving high performance on improving population health. The argumentation suggests that achieving a high-performance, sustainable, and impactful health organization can be translated into positively impacting population health with financial accountability, and systems internal processes may serve as roads to achieve that impact on society.

 At the original concept of value-based healthcare (VBHC), Prof. Proter defined what it means to build and implement the social science behind strategy for healthcare business.^1^ Having previously signed the most disseminated strategy literature and frameworks in history, such as Porter’s five forces,^2^ adding VBHC to the ecosystem also means matching previous strategy and performance literature to guide the building of sustainable and financially competitive organizations in healthcare business.

 Creating and managing competition is an elementary principle of developing strong businesses strategically. Competition stimulates innovation, efficiency, and the continuous search to deliver better experiences to customers, who become more demanding yearly.^3^ Bringing it to healthcare, a seminal paper named “Why Strategy Matters Now” published in the *New England Journal of Medicine* in 2015 by Proter and Lee^4^ requires our attention: it starts by clearly defining the differences between “operational effectiveness” and “strategy” and its implications to healthcare systems. Operational effectiveness is about improving what is being done, reducing errors and harms, and improving efficiency. Strategy involves making decisions and choices. Having an effective healthcare service can be measured by the hospital occupation rate, bed turn, or infection rates, while having a strategic health organization can be identified by the services that each organization offers, how patients identify this organization, and how they get into the service provided; how services are offered, how the hospital gets paid, and how the hospital pays for the providers.^5^ Merging the fields of strategy, performance, and value literature from Porter, it could be pointed out that designing health systems based on value involves setting a patient-based strategy that is a consequence of effective care, quality, and communication processes.

 Although the VBCH concept has been disseminated, the van Elten and colleagues’^6^ scoping review suggests that the current literature of applied VBHC cases does not demonstrate how to value projects generate impact at an organizational level and operational performance. The study also criticizes that one potential reason is that value measures are mainly focused on clinical issues, from the patient’s perspective, the costs associated with providing those outcomes, and not necessarily operational issues. However, in the value literature, it is understood that achieving good outcomes from the patient’s perspective with financial accountability is also a consequence of delivering effective processes; once it is being measured, the results are felt by customers (patients) and the costs to generate those results.

 This raises the question that healthcare leaders should consider, and is the main focus of this commentary paper: *What does it mean to have a high-performance, sustainable, and impactful health organization?*

 The value literature, as well as the health economics and outcomes research field, clearly point out that structuring more effective healthcare systems means increasing population health.^7^ This involves structuring strong primary care, increasing access to health technologies, coordinating the care process considering patients’ clinical conditions, and ensuring financial accountability.^7^ Making it happen is a “team-game” and a transformative cultural change.^5^ It involves all the stakeholders: patients, providers, payers, manufacturers, and policy-makers. Because of these multi-sectorial and innovative characteristics, the system’s capabilities required to make it happen include generating social capital and following methods that allow the evaluation of the implementation process and the impact that the initiatives are causing, not only from a performance perspective, but also from clinical and financial outcomes for all the stakeholders involved on the path.^5^

 Lee defined in his recent book that social capital is generated by creating social networks and using them to improve what we do.^5^ The author suggests that by building social capital, health organizations do more for patients with high reliability and earn loyalty across colleagues. It means creating real connections based on trust across teams and organizations. In the healthcare environment, where care pathways require integration between teams and, sometimes, centers, effective communication and action, and continual evidence-based updating, social capital is a required ability to be developed by institutions that are targeting value creation based on excellence. It is complementary to the traditional performance evaluation models; it includes the human perspective, which is so intense in the healthcare business.

 Once the integration across teams and organizations is natural, a purpose that aligns them for the same target, followed by methods to make it happen, is how creating value in healthcare can become a measurable and structured process. Frameworks such as the LEADER, proposed by de Silva Etges et al,^8^ allow organizing the sequence of activities on cultural transformation projects, such as the VBHC initiatives in general. Since the early stage of a project, those frameworks introduce a governance plan, which will become how it will be possible to evaluate the implications that the initiative is causing in clinical outcomes, costs, and organizational performance.

 The practical implications of applying this structured orientation have contributed to the scale of the applications of VBHC worldwide, which, although emergent, already have histories to tell. At the conference offered by the International Consortium for Health Outcomes Measurement (ICHOM) in 2024, for example, 112 applied abstracts were presented, which are available online (https://conference.ichom.org/abstracts), and most of them report applied cases of value initiatives from 26 countries around the world. The case presented, which was awarded as the best reported eleven years of ICHOM data collection at a private hospital in Brazil and the implications of this initiative in readmission and hospitalization reduction, which results in cost savings and operational wins. Another case from the Netherlands demonstrated how, by implementing a digital solution to monitor patient-reported outcomes, it was possible to reduce 90% of hospital visits and 60% of hospitalizations of patients under peritoneal dialysis. Those pieces of evidence demonstrate the indirect implications of value initiatives in operational settings. They are examples of making choices, implementing strategic projects, considering the value principles, and achieving operational and clinical wins.

 Understanding the impact of those initiatives worldwide, the ISPOR Society has launched a specific Task Force (https://www.ispor.org/member-groups/task-forces/value-based-healthcare-implementation) to define its position on the topic. At the last three ISPOR conferences, the group behind the Task Force presented how it is merging the concepts of health economics and outcomes research and VBHC to improve decision-making processes in healthcare, aiming to center care on patients’ needs, respecting the budgetary limitations and operating with excellence along with all the levels of healthcare systems. In the seminal concepts of value^1^ and health economics,^9^ there is a clear consensus about implementing more efficient processes to create more sustainable health systems globally. In both fields, measuring efficiency involves integrating the process and evaluating the implications that strategies are causing to society’s health, assuming that for this, evaluating clinical implications, from the patient’s perspective, is the central point, followed by the financial and operational performance metrics that should be implemented and managed.

 The World Health Organization (WHO)^10^ has also published advances on the VBHC topic by joining several applied cases from high-income and low-income countries stratified on the value-agenda framework. In addition to providing examples, the document also contributes in suggesting two elementary aspects behind impactful value initiatives: (*i*) the importance of integrating value with the health technology assessment field and how it is necessary to introduce more sustainable and effective pricing and reimbursement strategies for the health technologies; and (*ii*) the importance of establishing strong leadership across multiple stakeholders to implement healthcare transformations, such as those that consider the principles of value and aims to change how healthcare services are structured, delivered, paid, and effectively contribute to population health.

 Based on the background presented and the position of essential institutions from healthcare ecosystems, achieving a high-performance, sustainable, and impactful health organization can be translated into positively impacting population health with financial accountability. The applied examples that start to emerge demonstrate that the choice to implement VBHC is becoming more expressive not only in the United States or European Countries, but as is possible to observe in the ICHOM and ISPOR initiatives and WHO report, Latin Americans and Asian countries have demonstrated evidence of how by implementing value concepts involving multi-stakeholders and aiming to integrate care pathways, financial, clinical, and operational results are being achieved. Consolidating methods to evaluate the impact that those strategies have can consider the traditional frameworks to assess performance from the operational management literature, whereas it can be added to its essence implications that the initiatives are causing patients’ opinions about their own health and clinical outcomes. After all, focusing on positively impacting population health with financial accountability may be the purpose of any organization that works in the healthcare ecosystem, and implementing effective processes can serve as roads to achieve that impact on society.

## Ethical issues

 Not applicable.

## Conflicts of interest

 Author declares that she has no conflicts of interest.

## References

[1] Porter ME (2010). What is value in health care?. N Engl J Med.

[2] Porter ME (2008). The five competitive forces that shape strategy. Harv Bus Rev.

[3] Porter ME (1997). Competitive strategy. Meas Bus Excell.

[4] Porter ME, Lee TH (2015). Why strategy matters now. N Engl J Med.

[5] Lee TH. Social Capital in Healthcare: How Trust and Teamwork Drive Organizational Excellence. John Wiley & Sons; 2025.

[6] van Elten HJ, Howard SW, De Loo I, Schaepkens F (2023). Reflections on managing the performance of value-based healthcare: a scoping review. Int J Health Policy Manag.

[7] de Silva Etges AP, Liu HH, Jones P, Polanczyk CA (2023). Value-based reimbursement as a mechanism to achieve social and financial impact in the healthcare system. J Health Econ Outcomes Res.

[8] da Silva Etges AP, Polanczyk CA, Nabi J (2024). Revitalizing stroke care: the LEADER strategy for sustainable transformation in health care delivery. Stroke.

[9] Drummond MF, Sculpher MJ, Claxton K, Stoddart GL, Torrance GW. Methods for the Economic Evaluation of Health Care Programmes. Oxford University Press; 2015.

[10] World Health Organization (WHO). A Global View of Value-Based Care: Theory Practices and Lessons Learned. WHO; 2025. https://www.who.int/publications/i/item/9789290220121.

